# Inferior Alveolar Intraneural Cyst Arising from Temporomandibular Joint

**DOI:** 10.5334/jbsr.2970

**Published:** 2022-11-22

**Authors:** Catherine Mignon, Pierre-Antoine Poncelet

**Affiliations:** 1 CUSL, BE; 2 Grand Hôpital de Charleroi, BE

**Keywords:** Intraneural cyst, temporomandibular joint, inferior alveolar nerve

## Abstract

**Teaching Point:** Cystic nerve enlargement near a joint should evoke an intraneural synovial cyst and lead to the tracking of the articular connection for confirmation, where ever the location within the body is.

## Case

**Figure 1:**
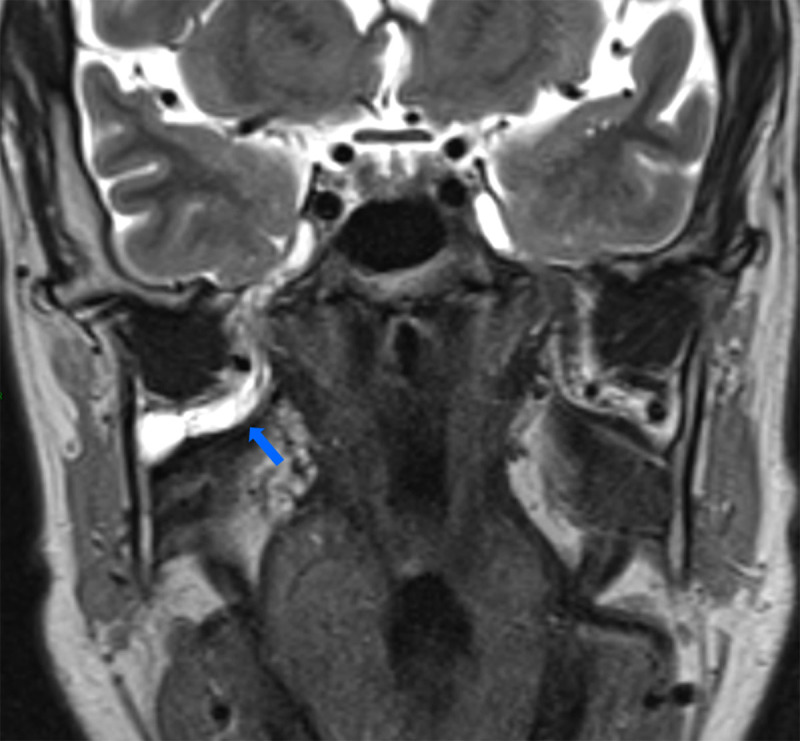


**Figure 2:**
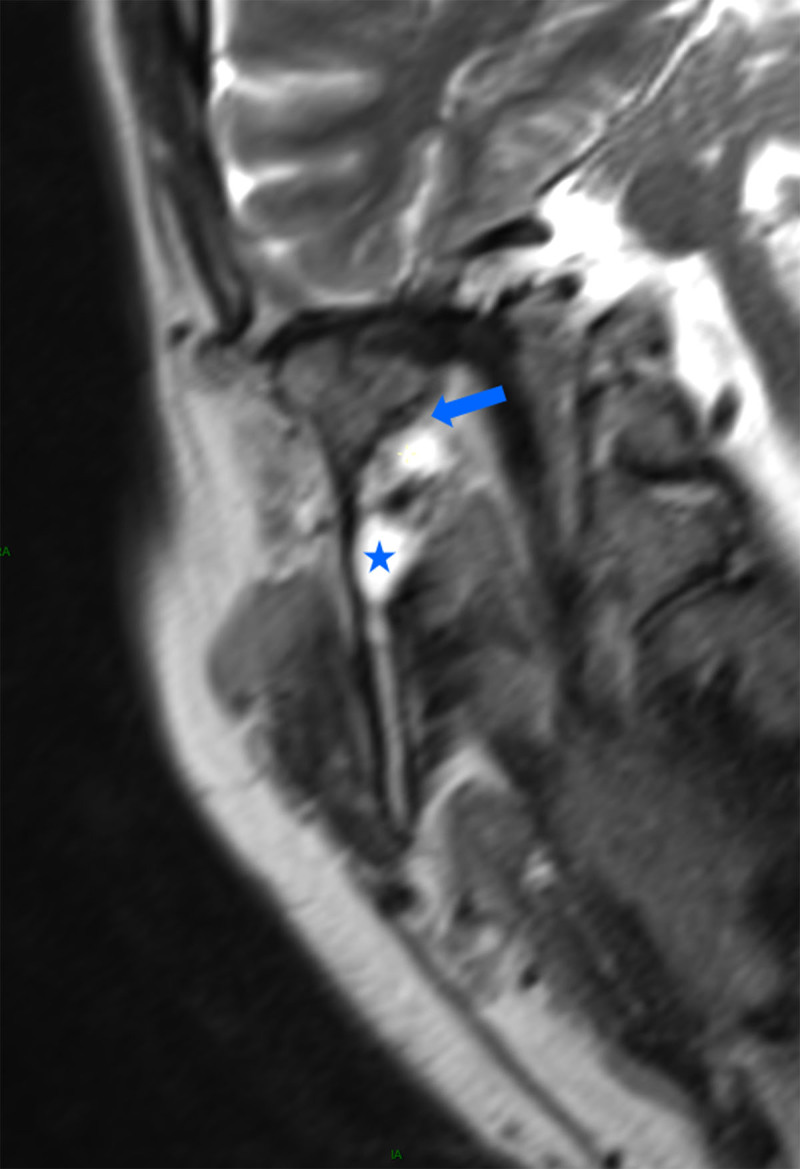


A 55-year-old male was referred to our radiology department to perform a head and neck MRI as part of the staging of a palatine tonsil squamous cell carcinoma with a necrotic adenopathy (not shown). Besides the tumor and the adenopathy, the MRI scan showed an ipsilateral bilocular cystic lesion within the masticator space running along the V3 nerve (Figure 1, arrow). The lesion was spreading from the foramen ovale to the mandibular foramen (Figure 1) and hugging the condylar process of the mandible on the oblique coronal reformatting slices (Figure 2, arrow). The mandibular foramen was enlarged by the lesion (Figure 2, star).

**Figure 3:**
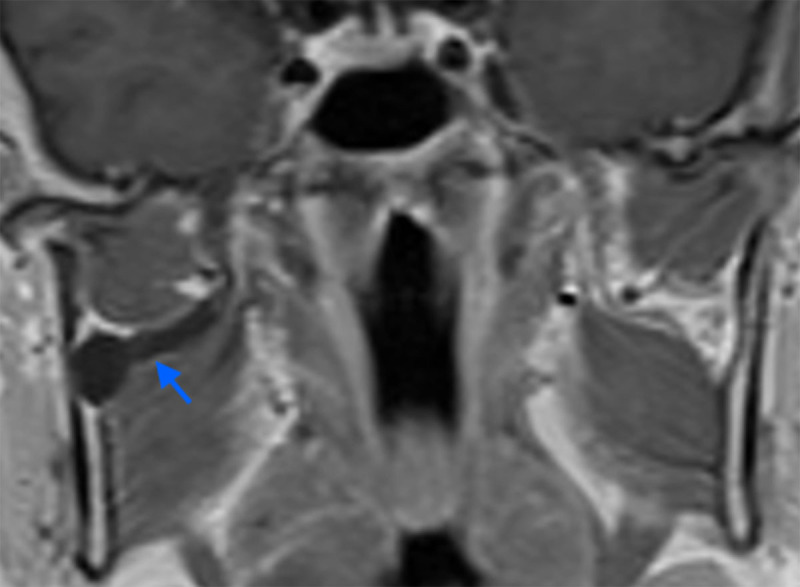


There was no enhancement of the pure cystic lesion (Figure 3, arrow), nor contact of the lesion with the tumor, excluding differential diagnosis such as cystic schwannoma, perineural spread of tumor or perineural lymphoma. These radiological features and its proximity with the temporomandibular joint highly suggested the diagnosis of an intraneural cyst of the inferior alveolar nerve.

The patient wasn’t symptomatic so therapeutic abstention was chosen.

## Discussion

Intraneural synovial cysts (also called ganglion cysts) are rare benign mucinous cysts contained within the external sheet of peripheral nerves. Several studies have recently provided evidence for an articular (synovial) pathogenesis: the cysts are formed from a capsular defect of a joint, propagate through the epineurium of an articular neural branch toward the parent nerve and their divisions. The cyst expansion depends on articular pressure and pressure fluxes [1]. It can lead to neuropathic symptoms or mass effects. Peripheral nerves are more frequently invaded by synovial cyst and especially the common peroneal nerve, but it has also been described for some cranial nerves: V3, VII and IX. Synovial cysts involving other cranial nerves have not been described in the literature. Interestingly, our case illustrates the first MRI images of a synovial cyst involving the inferior alveolar nerve. The cyst invades the articular branch of the auriculotemporal nerve and then goes on the mandibular division of the V and invades the inferior alveolar nerve [1].

Close examination of the images is essential and may permit to identify the articular connection of the cyst, which is often very thin. Contrast medium injection in the nearby articulation followed by a CT-scan or an MRI showing propagation in the cyst confirms the articular origin. Disconnection of the articular branch of the cyst is the main principle of the surgical management of the pathology, and statistically helps to prevent recurrence.

Although very rare in the head and neck region, ganglion cysts could be a clinically relevant pitfall in oncologic treatment planning mistaken for perineural tumor spread or missed as a potentially treatable cause of trigeminal neuropathy.
